# The use of climate information to estimate future mortality from high ambient temperature: A systematic literature review

**DOI:** 10.1371/journal.pone.0180369

**Published:** 2017-07-07

**Authors:** Michael Sanderson, Katherine Arbuthnott, Sari Kovats, Shakoor Hajat, Pete Falloon

**Affiliations:** 1 Met Office, Exeter, United Kingdom; 2 Faculty of Public Health and Policy, London School of Hygiene and Tropical Medicine, London, United Kingdom; 3 Centre for Radiation, Chemical and Environmental Hazards, Public Health England, Didcot, United Kingdom; Universidade de Vigo, SPAIN

## Abstract

**Background and objectives:**

Heat related mortality is of great concern for public health, and estimates of future mortality under a warming climate are important for planning of resources and possible adaptation measures. Papers providing projections of future heat-related mortality were critically reviewed with a focus on the use of climate model data. Some best practice guidelines are proposed for future research.

**Methods:**

The electronic databases Web of Science and PubMed/Medline were searched for papers containing a quantitative estimate of future heat-related mortality. The search was limited to papers published in English in peer-reviewed journals up to the end of March 2017. Reference lists of relevant papers and the citing literature were also examined. The wide range of locations studied and climate data used prevented a meta-analysis.

**Results:**

A total of 608 articles were identified after removal of duplicate entries, of which 63 were found to contain a quantitative estimate of future mortality from hot days or heat waves. A wide range of mortality models and climate model data have been used to estimate future mortality. Temperatures in the climate simulations used in these studies were projected to increase. Consequently, all the papers indicated that mortality from high temperatures would increase under a warming climate. The spread in projections of future climate by models adds substantial uncertainty to estimates of future heat-related mortality. However, many studies either did not consider this source of uncertainty, or only used results from a small number of climate models. Other studies showed that uncertainty from changes in populations and demographics, and the methods for adaptation to warmer temperatures were at least as important as climate model uncertainty. Some inconsistencies in the use of climate data (for example, using global mean temperature changes instead of changes for specific locations) and interpretation of the effects on mortality were apparent. Some factors which have not been considered when estimating future mortality are summarised.

**Conclusions:**

Most studies have used climate data generated using scenarios with medium and high emissions of greenhouse gases. More estimates of future mortality using climate information from the mitigation scenario RCP2.6 are needed, as this scenario is the only one under which the Paris Agreement to limit global warming to 2°C or less could be realised. Many of the methods used to combine modelled data with local climate observations are simplistic. Quantile-based methods might offer an improved approach, especially for temperatures at the ends of the distributions. The modelling of adaptation to warmer temperatures in mortality models is generally arbitrary and simplistic, and more research is needed to better quantify adaptation. Only a small number of studies included possible changes in population and demographics in their estimates of future mortality, meaning many estimates of mortality could be biased low. Uncertainty originating from establishing a mortality baseline, climate projections, adaptation and population changes is important and should be considered when estimating future mortality.

## Introduction

Warming of the Earth’s climate is now unequivocal; global average temperatures have risen by 0.85°C between 1880 and 2012 [1]. Increases in temperature over land areas are almost always higher than global average increases but vary between different regions of the Earth [2,3]. The frequency of heat waves has also increased in many continents [1]. Global mean temperature is projected to increase by about 1.6 to 2.6°C above the preindustrial period by the 2050s, depending on the scenario used [1]. Using median values, projected temperature increases for Europe and America are between 2 and 4°C for the 2050s (relative to present-day climate). Higher increases are projected over much of Asia and Australia [3].

There is increasing concern over the effects of hot weather on public health, including heat-related mortality and morbidity [4]. Deaths from high temperatures and heat waves are greater than deaths from other weather events such as tornados and flooding [5]. These deaths are not only a result of heatstroke. Existing studies generally examine the relationship between short-term fluctuations in temperature and all-cause (or cause specific, e.g. cardiovascular) mortality. The first studies linking mortality to warm temperatures were published in the early twentieth century [6]. Since this time, there have been numerous additional studies of the effects of specific periods of warm and hot weather on mortality, many of which have been reviewed elsewhere [7,8]. More recently, heat-related mortality has gathered increased attention in public health research owing to the acceptance that the Earth’s climate is warming and the large number of deaths caused by extreme heat waves (for example, Europe, 2003, 2015; Russia, 2010; Australia, 2012/2013 and 2016/2017; North America, 2012; India and Pakistan, 2015). Some of these events have led to the implementation of specific policies to reduce heat-related mortality such as the National Heat Wave Plan in France [9] and the Heatwave Plan for England [10].

Excess mortality from high temperatures has been reported in the first five assessment reports published by the Intergovernmental Panel on Climate Change (IPCC) to varying degrees. In the first report [11] the effects of warm temperatures and heat waves on mortality were briefly discussed. It was speculated that mortality from heat waves was likely to increase under a warming climate. The second [12], third [13], fourth [14] and fifth [15] assessment reports each contain a chapter devoted to human health. Mortality from high temperatures was discussed briefly in each report, but few studies were cited and the methods used to estimate future mortality were not assessed. Fourteen studies of projections of future heat related mortality have been reviewed previously [5], but these authors did not critically review the use of climate model data.

The aim of the present study is to review the use of climate model data in projections of future heat-related mortality. Morbidity was not considered; there are very few papers projecting future morbidity, and the burdens are very dependent on changes in health care. An important aspect of the present review is the critical appraisal of the selection of climate data and its use, and the methods employed to combine climate model data with observations. The treatment of uncertainty in climate model projections is also assessed. The epidemiologic models used to relate mortality to temperature and other variables have been reviewed elsewhere [5,8,16] and will not be addressed in the present study.

## Methods

### Data sources and search strategy

The peer-reviewed databases Web of Science and PubMed/Medline were searched with a focus on the titles of the articles; trial searches using a more general search of topics identified many hundreds of articles, most of which were irrelevant. Groups of two or three of the following keywords were used in the searches: mortality, future, climate, climate change, impacts, projection, heat, temperature, deaths and scenario. The search was limited to papers published in peer-reviewed journals in English with no restriction on year up to the end of March 2017. It is noted that the Web of Science does not contain articles published before 1981.

### Inclusion criteria

Two criteria were used to select articles for further study. The articles had to include at least one quantitative estimate of future heat-related mortality. Studies which only reported changes in morbidity, mortality resulting from air pollution or infectious diseases, or focused on winter and the effects of cold temperatures were not selected. Conference abstracts, books and publications by governments or international organisations were not included. Reference lists in the articles selected, and those studies which cited them were examined to ensure no relevant publications had been missed. Two of the authors independently examined the titles and abstracts of the articles identified in the searches of the databases to assess their relevance. A protocol for this systematic review has not been published.

### Quality assessment

There is no accepted standard procedure for assessing the quality of climate models and their data, although some recommendations have been made [17,18]. The risk of bias was assessed in two domains (spread in projections amongst climate models and emissions scenarios used). The study quality was assessed by one reviewer.

### Data synthesis

A meta-analysis of the results was not conducted. The locations, time periods studied and climate model data used to estimate future heat-related mortality varied considerably between the selected articles. Instead, a descriptive summary of the estimates is provided.

## Results

**Fig 1:**
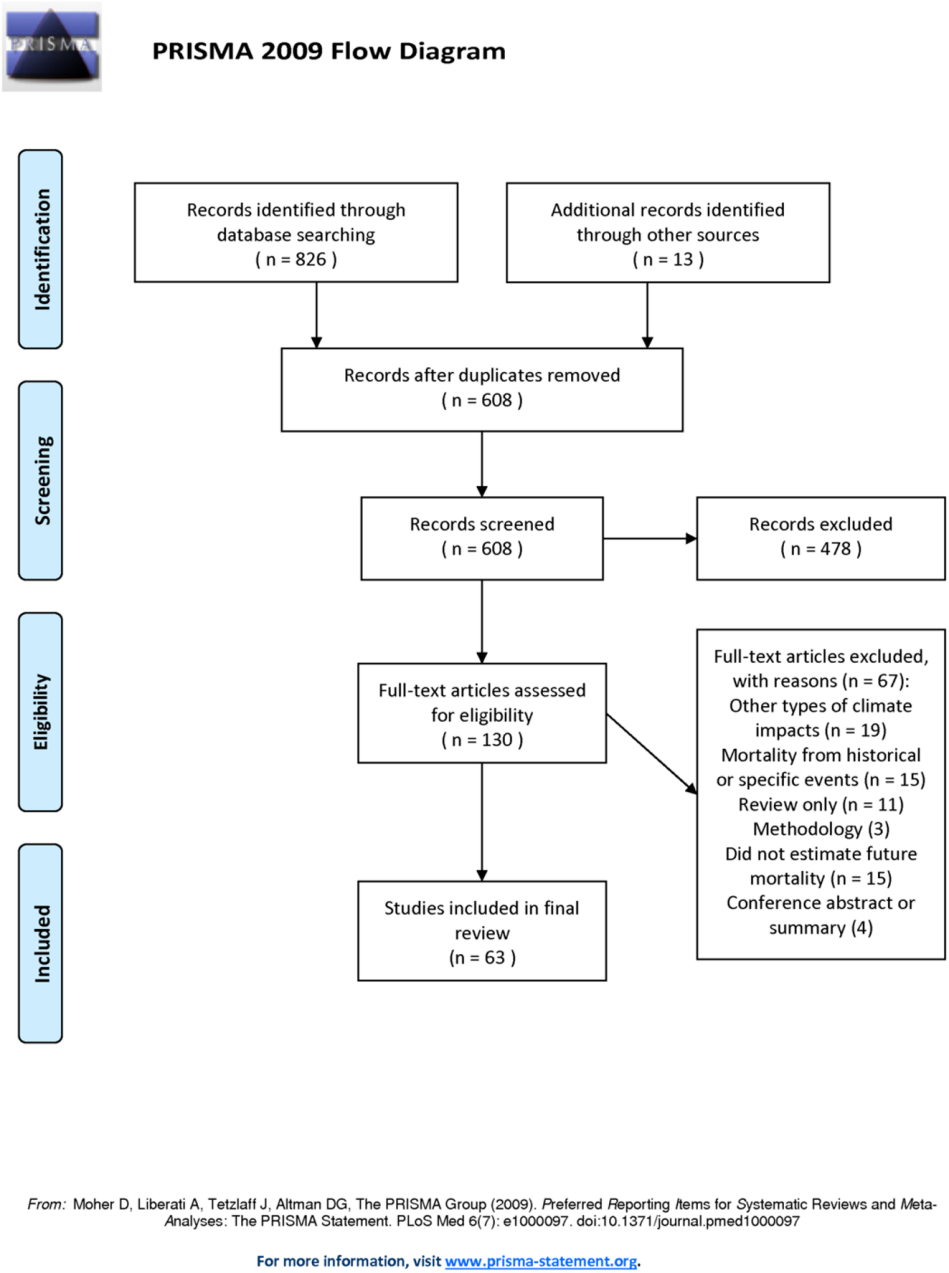
Flow chart illustrating the process of article selection and rejection following the PRISMA guidelines.

Initially, 608 articles were identified from the literature searches after duplicate entries had been removed. Thirteen additional articles were selected from examination of reference lists and citing literature. After screening the titles and abstracts, the full texts of 130 articles were examined in detail. Of these articles, 63 were found to contain quantitative estimates of future heat-related mortality and so were selected for the systematic review (Fig 1). The locations studied, time periods, climate models, emissions scenarios and treatment of adaptation are summarised in Table 1, together with the meteorological variables used in each study. Further technical details of each study, specifically the variables used to model mortality, calibration methods, time of year considered and consideration of changes in population are shown in Table 2. The locations of the cities studied are shown in S1 Fig, and the time periods considered in each article are illustrated in S2 Fig.

**Table 1:** Summary of the 63 studies which included a quantitative estimate of future heat-related mortality.

Article and Reference	Location	Study periods	Global Models (number used). Scenarios (number analysed).	Downscaling Method (No. simulations). Resolution of RCM.	Total no. sims	Met Var(s)	Adaptation Method
Baaghideh and Mayvaneh (2017) [63]	Mashhad, Iran	Obs: 2004–2013 Baseline: 1986–2005 Future: 2021–2099	GCM (1) A2	WG	1	TX	None
Petkova et al. (2017) [118]	New York	Obs: 1900–2006 Baseline: 1970–1999 Future: 2010–2039, 2040–2069, 2070–2099	GCM (33) RCP4.5, RCP8.5	BCSD (⅛°)	66	TM	Rel
Li et al. (2016) [105]	Beijing	Obs: 2008–2011 Baseline: 1970–1999 Future: 2010–2039, 2040–2069, 2070–2099	GCM (31) RCP4.5, RCP8.5	BCSD (0.5°)	62	TM	Rel, slope
Lee and Kim (2016) [36]	7 cities in South Korea	Obs: 1992–2010 Future: 2000–2100	GCM (1) RCP2.6, RCP4.5, RCP6.0, RCP8.5	Not stated	4	TM	None
Heaviside et al. (2016a) [68]	Nicosia and Cyprus	Obs: 2004–2009 Baseline: 2004–2009 Future: ~2010–2100	Fixed T (1–5°C)	None	5	TX	Abs (+1.2°C)
Roldán et al. (2016) [96]	Zaragoza (Spain)	Obs: 1987–2006 Baseline: 1987–2006 Future: 2014–2021	GCM (1) A2, A1B, B1	Stat, daily.	3	TX	None
Martinez et al. (2016) [54]	Skopje	Obs: 1986–2005 Baseline: 1986–2005 Future: 2026–2045, 2081–2100	GCM (3) RCP8.5	RCM (3), 250 m	3	TM	None
Gosling et al. (2016) [46]	14 European cities	Obs: 1958–2001 Baseline: 1981–2010 Future: 2070–2099	GCM (5) RCP2.6 (1), RCP8.5 (5)	Stat, daily (0.5°).	6	TX, TM, RH	6 different methods
Heaviside et al. (2016b) [55]	West Midlands, UK	Obs: 1–10 Aug 2003 Baseline: 1961–1990 Future: 2010–2039, 2040–2069, 2070–2099	A1FI, A1B, B1	RCM§ 25 km	3	TM	None
Kingsley et al. (2016) [57]	Rhode Island	Obs: 1999–2011 Baseline: 2005–2012 Future: 2046–2053, 2092–2099	GCM (42) RCP4.5 (42), RCP8.5 (41)	BCCA (^1^/_8_°)	83	TX	None
Guo et al. (2016) [62]	3 cities in Australia	Obs: 1988–2009 Baseline: 2000–2009 Future: 2050s, 2090s	GCM (62). A2 (18), A1B (23), B1 (21)	Stat, monthly. WG, daily.	62	TX, RH	None
Kim et al. (2016) [35]	Korea	Obs: 1994–2012 Future: 2013–2060	GCM (1). RCP4.5, RCP8.5	RCM (1), 12.5 km. Stat to 1 km.	2	TX	None
Huynen and Martens (2015) [53]	The Netherlands	Obs: 1981–2010 Baseline: 1981–2010 Future: 2035–2065	KNMI’14 (4)	RCM (1), plus stat to individual sites.	4	TM	Abs; slope
Li et al. (2015) [112]	Beijing	Obs: 1971–2000 Baseline: 1971–2000 Future: 2010–2039, 2040–2069, 2070–2099	GCM (5). RCP4.5, RCP8.5	BCSD (^1^/_8_°)	10	TM	None
Murari et al. (2015) [37]	4 states in India	Obs: 1970–1999 Baseline: 1970–1999 Future: 2010–2039, 2040–2069, 2070–2099	GCM (7). RCP2.6, RCP4.5, RCP8.5	Bilinear interpolation to regular 1° grid	21	TX, vapour pressure	None
Schwartz et al. (2015) [101]	209 cities in the USA	Obs: 1976–2005 Baseline: 1976–2005 Future: 2016–2045, 2036–2065, 2086–2100.	GCM (2). RCP6.0	BCCA (1°)	2	TM	None
Mills et al. (2015) [22]	33 cities in the USA	Obs: 1980–2009. Baseline: 1999–2001 Future: 2049–2051, 2099–2101.	GCM (1). REF, POL3.7	None	2	TN	Abs (max threshold from all cities)
Zacharias et al. (2015) [95]	Germany	Obs: 2001–2010 Baseline: 1971–2000 Future: 2021–2050, 2069–2098	A1B	RCM (19). 10 km, 25 km	19	TM	Rel (50%)
Zhang et al. (2014) [33]	3 cities in China	Obs: 2001–2008 Future: 2080–2099	Fixed T (1, 2, 3, 4°C)	None	4	TM	None
Benmarhnia et al. (2014) [84]	Montreal, Canada	Obs: 1990–2007 Baseline: 1990–2007 Future: 2020–2037	GCM (4): A2 (7), A1B (8), B1(7)	RCM (1). A2 (10) 45 km.	32	TX, TM, TN	None
Vardoulakis et al. (2014) [25]	England, Wales, Australia‡	Obs: 1993–2006 Future: 2020–2029, 2050–2059, 2080–2089	A1FI, A1B, B1	RCM§ 25 km	3	TM	None
Jenkins et al. (2014) [61]	Greater London	Obs: 1961–1990 Baseline: 1961–1990 Future: 2020–2049, 2040–2069	A1FI, B1	RCM§ 25 km. WG.	2	TM	Abs (1°C, 2°C)
Petkova et al. (2014) [110]	12 cities in USA	Obs: 1987–2005 Baseline: 1970–1999 Future: 2010–2039, 2040–2069, 2070–2089.	GCM (16). A2, B1	BCSD (^1^/_8_°)	32	TM	None
Bobb et al. (2014) [66]	105 cities in USA	Obs: 1987–2005 Future: ~2040–2059	Fixed T (5°F = 2.8°C)	None	1	TM	None
Wu et al. (2014) [31]	Eastern USA	Obs: 2001–2004 Baseline: 2001–2004 Future: 2057–2059	GCM (1). RCP4.5, RCP8.5	RCM (1) 4 km	2	TX, TM, TN	None
Hajat et al. (2014) [29]	UK	Obs: 1993–2006. Baseline: 2000–2009; Future: 2020–2029, 2050–2059, 2080–2089	GCM. A1B (9)	RCM (9) 25 km	9	TM	None
Honda et al. (2014) [45]	WHO regions (global)	Obs: 1972–2008 Baseline: 1961–1990 Future: 2030, 2050	GCM (1). A1B	None	1	TX	Rel
Tawatsupa et al. (2014) [32]	Thailand	Obs: 1999–2008 Future: ~2100	Fixed T (4°C)	None	1	TX	None
Kim et al. (2014) [34]	Six cities in Korea	Obs: 2001–2008 Baseline: 2001–2010 Future: 2041–2070, 2071–2100	GCM (1). RCP4.5, RCP8.5	RCM (1), then stat to 1 km(?)	2	TM	None
El Fadel and Ghanimeh (2013) [89]	Beirut	Obs: None Baseline: 1961–1990 Future: 2010–2050, 2050–2095	GCM (1): A2, A1FI, B1	RCM (2). A1B 30 km	5	TM	Abs (1°C)
Li et al. (2013) [111]	New York	Obs: 1982–1999 Baseline: 1980–1999 Future: 2010–2039, 2040–2069, 2070–2089	GCM (16). A2, B1	BCSD (^1^/_8_°)	32	TX	None
Petkova et al. (2013) [109]	3 cities in the USA	Obs: 1985–2006 Baseline: 1971–2000 Model: 2010–2039, 2040–2069, 2070–2099	GCM (33). RCP4.5, RCP8.5	BCSD (^1^/_8_°)	66	TX, TM, TN	None
Barreca (2012) [71]	350 counties in the USA	Obs: 1968–2002 Future: 2070–2099	GCM (1). A1FI	IDW	1	TM, SH	None
Martin et al. (2012) [54]	15 cities in Canada	Obs: 1981–2000 Baseline: 1981–2000 Future: 2031–2050, 2051–2070, 2071–2090	GCM (1). A2	RCM (1). 45 km.	1	TM	None
Morabito et al. (2012) [43]	10 cities in Tuscany.	Obs: 1999–2008. Baseline: 1999–2008 Future: 2011–2030, 2031–2050	GCM (1). A1B	RCM (1), 50 km. WG	1	TM	None
Sheridan et al. (2012) [44]	Nine urban locations in California.	Obs: 1975–2004 Future: 2000–2099	GCM (2). A1FI (1), A2 (2), B1 (2).	None	5	SSC weather types	Ignored mortality in first 3 days
Gosling et al. (2012) [88]	Boston, Budapest, Dallas, Lisbon, London, Sydney	Baseline: 1961–1990 Future: 2070–2099	GCM (18). A2 (1), A1B (18), B1 (1)	RCM (11)§, 25 km. A1B.	31	TX	None
Zhou et al. (2012) [92]	Three cities in Alabama	Obs: 1991–2000 Baseline: 2000 Future: 2041–2050	GCM (1). A2	RCM (1) 50 km.	1	TX	None
Ostro et al. (2012) [70]	4 cities in Catalonia (north east Spain).	Mortality: 1983–2006. Baseline: 1961–1990 Future: 2010–2040, 2035–2065	GCM (4). A1B	RCM (8), 25 km; IDW.	8	TM	None
Watkiss and Hunt (2012) [108]	EU-27	Baseline: 1961–1990 Future: 2011–2040, 2071–2100	GCM (3). A2 (3), B2 (2)	RCM (2), 50 km. A2 (3), B2 (2)	5	TM	Abs (+1°C per 30 years)
Deschênes and Greenstone (2011) [72]	USA	Obs: 1968–2002. Baseline: 1968–2002 Future: 2070–2099	GCM (2). A1FI (1), A2 (1)	None	2	TM	None
Ballester et al. (2011) [78]	16 European countries	Obs: 1998–2003. Model: 1950–2100.	GCM (5). A1B	RCM (8), 25 km	8	TM, RH	Abs
Ostro et al. (2011) [30]	California	Mortality: 1999–2007 Baseline: 1961–1990 Future: 2024–2026, 2049–2051.	GCM (2). A2 (1), B1 (1)	BCSD (^1^/_8_°)? Stated that daily data were used (BCCA?)	2	TM, RH	Slope
Peng et al. (2011) [94]	Chicago	Obs: 1987–2005. Baseline: 1981–2000 Future: 2081–2100	GCM (7). A2, A1B, B1	None	7	TX	None
Voorhees et al. (2011) [77]	USA (entire)	Baseline: 1998–2003 Future: 2048–2052	GCM (1). A1B	RCM (1), 36 km.	1	TX, RH	None
Greene et al. (2011) [56]	40 large cities in the USA	Obs: 1975–2004. Baseline: 1975–1995 Future: 2020–2029, 2045–2055, 2090–2099	GCM (1). A1FI, B1	Stat	2	TX, TN, T_dew_ SSC weather types	Difference in mortality over two time periods
Baccini et al. (2011) [65]	15 European cities	Obs: 1990–2001 Baseline: 1980–1999. Future: 2030.	Fixed T (various)	None	3	TX, RH	None
Hayhoe et al. (2010) [76]	Chicago	Baseline: 1961–1990 Future: 2010–2039, 2040–2069, 2070–2099	GCM (3): A1FI (3); B1 (3)	Stat	6	TX, TN; SSC weather types.	None
Jackson et al. (2010) [79]	Four areas in Washington State	Obs: 1980–2006. Baseline: 1970–1999 Future: 30 year periods centred on 2025, 2045, 2085	GCM (2). A1B (1), B1 (1), plus average of the two scenarios.	None	3	HX	None
Muthers et al. (2010) [73]	Vienna	Obs: 1970–2007 Baseline: 1970–2000 Future: 2011–2040, 2041–2070, 2071–2100	GCM (1). A1B, B1	RCM (2). 10 km, 18 km. A1B (2), B1 (2)	4	PET	Extrapol of mortality trend
Gosling et al. (2009b) [42]	Six cities worldwide	Baseline: 1961–1990 Future: 2070–2099	GCM (1). A2, B2	None	2	TX	Abs (+2°C, +4°C)
Cheng et al. (2008) [107]	4 cities in Canada	Obs: 1954–2000; NCEP (1961–2000) Baseline: 1961–2000 Future: 2040–2059, 2070–2089.	GCM (3). IS92a (1), A2 (2), B2 (2)	Stat	5	TM	Hottest and coolest summers
Doyon et al. (2008) [129]	3 cities in Canada	Obs: 1981–1999 Baseline: 1981–1999 Future: 2010–2039, 2040–3069, 2070–2099.	GCM (1). A2 (1), B2 (1).	Stat	2	TM	None
Takahashi et al. (2007) [41]	Global	Obs: 1991–2000 Baseline: 1991–2000 Future: 2091–2100	GCM (1). A1B	None	1	TX	None
Knowlton et al. (2007) [69]	New York	Obs: 1993–1997 Baseline: 1993–1997 Future: 2053–2057	GCM (1). A2, B2	RCM (1), 36 km; IDW.	2	TM	Analogue cities
Hayhoe et al. (2004) [40]	Los Angeles	Obs: 1961–1990 Baseline: 1961–1990 Future: 2020–2049, 2070–2099	GCM (2): A1FI (2), B1 (2)	BCSD, to ⅛ °; then to station sites	4	TX, RH	Hottest summers
Dessai (2003) [91]	Lisbon, Portugal	Obs: 1980–1998 Baseline: 1969–1998 Future: 2020s, 2050s, 2080s.	GCM (1). 2 × CO2.	RCM (2), ~50 km.	2	TX	Abs (+1°C per 30 years)
Guest et al. (1999) [87]	5 cities in Australia	Obs: 1979–1990 Baseline: 1979–1990 Future: 2024–2035	GCM (1). 2 × CO2, scaled by global mean warming.	None	1	TX, TSI weather types	None
Martens (1998) [75]	20 cities worldwide	Obs: 1961–1990 Baseline: 1961–1990 Future: ~2040–2100	GCM (3). Scenarios not stated.	None	3	TM	Slope
Kalkstein and Greene (1997) [86]	44 cities in USA	Obs: 1961–1990 Future: ~2020, ~2050	GCM (3), transient scenarios.	None	3	TX, TN, RH; SSC weather types;	Analogue cities
Kalkstein and Smoyer (1993) [85]	28 cities in USA, China, Canada and Egypt.	Baseline: Not stated Future: ~2060	GCM (1). 2 × CO2.	None	1	TX; TSI weather types	Hot and cold summers; slope
Kalkstein (1993) [67]	15 cities in the USA	Not stated	GCM (1). Transient and 2 × CO2; fixed T (2°C)	None	3	TX; TSI weather types	Not stated
Kalkstein (1988) [28]	15 cities in the USA	Obs: 1964–1966, 1972–1978, 1980 Baseline: Not stated Future: ~2040–2100	Fixed T, 2–7°F (~1.1–4.0°C)	None	5	TX, TM, TN	Analogue cities

The first two columns list the references and location(s) studied. *Study periods*–Obs refers to observations of mortality and local climate; baseline and future refer to model data. *Global Models / Scenarios*–GCM (n) indicates the number of global climate model simulations used. Scenarios: IS92a is one of six scenarios published in 1992 [19]. A1FI, A2, A1B, B1 and B2 are SRES scenarios [20]. RCP2.6, RCP4.5, RCP6.0 and RCP8.5 are representative concentration pathways [21]. REF and POL3.7 are similar to the RCPs but have different radiative forcings [22]. Numbers in parentheses indicate the number of simulations analysed which were generated using that particular scenario. In some cases a climate model was used multiple times with the same scenario, and only the initial conditions were changed. Fixed T means GCM data were not used. Instead, a temperature increase was prescribed. *Downscaling Method*–RCM means a regional climate model was used to dynamically downscale global climate model simulations. The number of simulations analysed is indicated in brackets; in some studies, multiple RCMs had been used to downscale the same GCM simulation. The resolution(s) of the RCM(s) is also given. Stat–the model results were statistically downscaled at the timescale indicated. WG means a weather generator was used to produce daily climate data. BCSD and BCCA are bias-corrected and statistically downscaled data at monthly and daily timescales respectively [23]. IDW means inverse distance weighting was used to interpolate climate model data to a specific point from surrounding grid boxes. *Total no*. *sims*—the total number of climate model simulations analysed in each study. *Meteorological variable(s)*–the variable(s) used to either model heat-related mortality or calculate other indices. TX, TM, TN are daily maximum, mean and minimum temperatures. RH and SH are relative and specific humidity. T_dew_ is the dew point temperature. *Adaptation Method*–the method(s) used to model adaptation of the population to warmer temperatures. Abs–the mortality threshold temperature was increased by a fixed amount; Rel–the mortality threshold was modified by applying the percentile of the threshold to future temperatures and then adjusting the threshold to be between these two limits; slope–the slope of the exposure-response function was reduced; analogue city–use of exposure-response functions for a city whose present-day temperatures are similar to those projected to occur at the location of interest in the future. “None” means adaptation was not considered.

§These studies used one or more of the probabilistic climate projections from the United Kingdom Climate Projections 2009 (UKCP09) [24].

‡The probabilistic projections for Australia used by Vardoulakis et al. [25], “OzClim”, were based on a large ensemble of GCM simulations. They have been superseded by a newer set of probabilistic projections.

**Table 2:** Technical details of observations used, calibration methods, months considered and population/demographic changes.

Article and Reference	Mortality variable(s)	Observations	Calibration Method (Time scales)	Months studied	Population / Demographics
Baaghideh and Mayvaneh (2017) [63]	TX	Weather Sta	Not stated	January—December	Constant
Petkova et al. (2017) [118]	TM	Weather Sta	Delta (monthly)	June—September	Pop + Dem
Li et al. (2016) [105]	TM	Weather Sta	Delta (monthly)	January—December	Pop (Age 65+ only)
Lee and Kim (2016) [36]	TM	Weather Sta	Not stated	January—December	Pop + Dem
Heaviside et al. (2016a) [68]	TX	Weather Sta	Delta (fixed T)	April—September	Pop
Roldán et al. (2016) [96]	TX	Weather Sta	Included in downscaling	June—September	Pop + Dem
Martinez et al. (2016) [54]	TM	ERA-I; Weather Sta	Bias-Corr (hourly)	May—September	Pop
Gosling et al. (2016) [46]	AT	WATCH [26], 0.5°	Bias-Corr (daily)	April—September	Constant
Heaviside et al. (2016b) [55]	TM	Weather Sta	Delta (monthly)	1–10 August	Pop
Kingsley et al. (2016) [57]	TX	Weather Sta	BCCA	April—October	Constant
Guo et al. (2016) [62]	TX; RH	Weather Sta	Quantile (monthly); Weather Generator	January—December	Constant
Kim et al. (2016) [35]	TX	Weather Sta	Statistical (daily)	July—August	Pop + Dem
Huynen and Martens (2015) [53]	TM	Weather Sta	Included in downscaling	January—December	Pop + Dem
Li et al. (2015) [112]	TM	Weather Sta	Delta (monthly)	January—December	Constant
Murari et al. (2015) [37]	Heat wave days	Gridded 1°; NCEP Reanalysis.	Quantile	March—May	Constant
Schwartz et al. (2015) [101]	TM	Weather Sta	Delta (daily)	April—September	Constant
Mills et al. (2015) [22]	TN	Weather Sta	Delta (daily)	May—September	Pop
Zacharias et al. (2015) [95]	TM	Weather Sta	Percentile	January—December	Constant
Zhang et al. (2014) [33]	TM	Weather Sta	Delta (fixed T)	January—December	Constant
Benmarhnia et al. (2014) [84]	TX; TM; TN	Weather Sta	Shift (daily)	June—August	Constant
Vardoulakis et al. (2014) [25]	TM.	Weather Sta (averaged over regions)	Delta (monthly)	June—September; December—March	Pop + Dem
Jenkins et al. (2014) [61]	TM	Weather Generator	Delta (monthly)	January—December	Pop + Dem
Petkova et al. (2014) [110]	TM	Weather Sta	Delta (monthly)	January—December	Constant
Bobb et al. (2014) [66]	TM	Weather Sta	Delta (fixed T)	June—August	Constant
Wu et al. (2014) [31]	TX; TM; TN; HI	Weather Sta (averaged over regions)	Multiplicative	May—September	Pop
Hajat et al. (2014) [29]	TM	Weather Sta (averaged over regions)	Percentile	January—December	Pop + Dem
Honda et al. (2014) [45]	TX	Reanalysis data corrected with gridded observations	Delta (monthly)	January—December	Pop
Tawatsupa et al. (2014) [32]	TX	Weather Sta (averaged over regions)	Delta (fixed T)	November—February; March—June; July—October	Constant
Kim et al. (2014) [34]	TM	Weather Sta	Percentile	June—September	Pop
El Fadel and Ghanimeh (2013) [89]	TM	None	Delta (annual)	January—December	Constant
Li et al. (2013) [111]	TX	Weather Sta	Delta (monthly)	January—December	Constant
Petkova et al. (2013) [109]	TX; TM; TN	Weather Sta	Delta (monthly)	May—September	Constant
Barreca (2012) [71]	TM; SH.	Weather Sta (averaged over regions)	None	January—December	Constant
Martin et al. (2012) [54]	TM	Weather Sta	Delta (monthly) in 5 year groups	June—August	Constant
Morabito et al. (2012) [43]	TM	Weather Sta, gridded (200 m)	Monthly change factors used with a weather generator	January—December	Constant
Sheridan et al. (2012) [44]	SSC	SSC	None	March—November	Pop + Dem
Gosling et al. (2012) [88]	TX	Weather Sta	Logistic distribution parameters	June—August (December—February for Sydney)	Constant
Zhou et al. (2012) [92]	TX	Weather Sta (averaged over regions)	Bayesian spatial quantile regression	May—September	Constant
Ostro et al. (2012) [70]	TM	Weather Sta	Percentile	15 May—15 October	Pop + Dem
Watkiss and Hunt (2012) [108]	TM	None	Percentile	January—December	Pop + Dem
Deschênes and Greenstone (2011) [72]	TM	Weather Sta (IDW over regions)	Shift (daily)	January—December	Constant
Ballester et al. (2011) [78]	AT; TM	Gridded (25 km) averaged over regions	Percentile	January—December	Constant
Ostro et al. (2011) [30]	AT	Gridded (~12 km)	BCSD	May—September	Pop + Dem
Peng et al. (2011) [94]	Heat waves (from TX)	Weather Sta	Ratios of heat wave lengths.	May—October	Pop + Dem
Voorhees et al. (2011) [77]	AT	None	None	May—September	Pop + Dem
Greene et al. (2011) [56]	SSC	Weather Sta	Shift (6 hourly)	June—August	Constant
Baccini et al. (2011) [65]	Daily max AT	Weather Sta	Delta (fixed T)	April—September	Constant
Hayhoe et al. (2010) [76]	AT and SSC	Weather Sta	Stat to 6 hourly	January—December	Constant
Jackson et al. (2010) [79]	HX	Gridded (^1^/_16_°) averaged over regions	Delta (monthly)	May—September	Constant (at 2025 levels)
Muthers et al. (2010) [73]	PET	Weather Sta	Percentile	April—October	Constant
Gosling et al. (2009b) [42]	TX	Weather Sta	Logistic distribution parameters	January—December	Constant
Cheng et al. (2008) [107]	TM	Weather Sta daily and 6 hrly. NCEP upper air reanalysis (daily)	Stat to hourly.	January—December	Constant
Doyon et al. (2008) [129]	TM	Weather Sta	Delta (monthly and annual)	January—December	Constant
Takahashi et al. (2007) [41]	TX	Gridded (0.5°)	Shift (monthly)	January—December	Constant
Knowlton et al. (2007) [69]	TM	Weather Sta; IDW to points	Delta (monthly)	June—August	Constant
Hayhoe et al. (2004) [40]	AT	Weather Sta	Quantile	January—December	Constant
Dessai (2003) [91]	TX	Weather Sta (?)	Delta (daily)	January—December	Pop
Guest et al. (1999) [87]	TX; TSI	Weather Sta (3 hourly)	Delta (monthly) scaled by global mean warming	“Summer”	Pop + Dem
Martens (1998) [75]	TM (monthly mean)	Weather Sta	Delta (monthly)	January—December	Constant
Kalkstein and Greene (1997) [86]	SSC; TX; TN; humidity	Weather Sta	None	June—August	Constant
Kalkstein and Smoyer (1993) [85]	TX; TSI	Weather Sta	Delta (monthly)	June—August	Constant
Kalkstein (1993) [67]	TSI	Weather Sta	Delta (fixed T)	June—August	Constant
Kalkstein (1988) [28]	TX; TM; TN; TSI	Weather Sta	Delta (fixed T)	June—August	Constant

The first column lists the reference for each study. *Mortality variables*–Variable(s) used for estimating mortality, daily values unless stated otherwise. TX, TM and TN are maximum, mean and minimum temperatures. AT is apparent temperature, WBGT is wet bulb global temperature, HI is the Humidex and HX is the Heat index. PET is physiologically equivalent temperature. AT, WBGT, HI, and HX are functions of temperature and humidity; PET is calculated with a separate model. SSC and TSI are synoptic-scale classifications of weather types. *Observations*–Type of observations used. “Weather Sta” indicates data from local or nearby weather stations were used. “Gridded” indicates data produced by applying a regression algorithm to surface-based observations to produce weather information on a regular grid with the stated resolution. IDW—inverse distance weighting was used to estimate weather data at a specific location from nearby stations. WATCH—The WATCH forcing data [26] were used in place of observations. None—no observations appear to have been used, and the study only considered modelled data. *Calibration method / Time Scales*–The calibration method by which observations and climate model data were combined and the timescales of the climate model data. Note that many studies combined monthly or annual change factors derived from climate model projections with observed daily or sub-daily data. Bias-Corr—a method which corrects the mean and variance [27] was used. BCCA / BCSD indicates bias-corrected and downscaled climate model data from [23] were used. *Months studied*–the range of months over which climate information was used to estimate heat-related mortality. *Population and Demographics*–whether the study included projected changes in population (“pop”) and/or demographics, specifically aging (“dem”) in their future mortality estimates. “Constant” means population numbers were held constant.

The first known attempt to estimate future heat-related mortality under a warming climate was published in 1988 [28]. Future mortality was estimated for fifteen cities in the USA using a range of prescribed temperature increases inferred from a single global climate model simulation. A small number of studies were published afterwards in the 1990s and early 2000s (Table 1). The number of studies of future heat-related mortality increased considerably after 2007, for which there are several possible reasons. Improved access to data from both global and regional climate model simulations around the same time could be one reason. Improved epidemiological methods, easier access to and speed of the Internet, and increased computational power of researchers’ workstations might be other factors.

### Locations studied

The majority of the locations studied are cities in high income countries including Canada, USA, European countries, South Korea and Australia (Table 1). The locations and numbers of studies which estimated future mortality for each city are shown in S1 Fig. There are no studies specifically of rural areas, although two studies calculated future mortality in different regions of the UK which included both urban and rural populations [25,29]. Two other studies [30,31] considered both rural and urban areas in parts of the USA. There are very few or no projections of future mortality for populations in Africa, the Middle East, South America, and much of northern, central and southern Asia. The only studies of future mortality in tropical and sub-tropical areas are those for Thailand [32], China [33], Korea [34,35,36] and India [37]. In some countries, complete mortality records may not be routinely available which limits epidemiological analyses.

### Observations of local weather and climate

All of the selected studies have either used local observations of weather variables and mortality records to construct suitable mortality models, or used mortality functions from previous studies. Very few cities contain dense weather observation networks. These networks tend to be organised for specific research projects and only exist for short time periods (for example, [38]). Many studies of mortality within cities have therefore used observations from nearby airports or parks within the city. Temperatures at these locations may not be representative of conditions in other parts of the city [39]. Estimates of the number of days above a threshold temperature could therefore be over- or underestimated in some city areas.

Some studies averaged observations within a given area to produce mean values for that area [29,27,31]. Given the sparseness of surface observations, these estimates may not represent the “true” area- averaged temperatures. Other studies used gridded temperature data which had been created by use of a regression model to interpolate irregularly spaced weather observations onto a regular grid [37,40,41,42,43]. These gridded datasets provide useful estimates of climate information for locations where surface-based observations are unavailable. Their accuracy depends on the number of weather stations available and the predictors used (for example, altitude, proximity to the coast, local topographical features). Some important climatic effects, such as the urban heat island, may not be included leading to underestimates of temperature in the gridded data at urban locations. Many observations contain measurement and sampling errors, but the magnitude of these errors is not always known. These errors are likely to be small compared with other sources of uncertainty.

A few studies have used climate data from reanalyses to supplement surface observations [37,44,45,46]. Global reanalyses are created by assimilating observations every 3–12 hours within a weather forecast model to provide a dynamically consistent description of the atmosphere. Global reanalyses available at the time of writing have spatial scales of the order of 30–180 km. In one study [45], climate variables from a global reanalysis were further downscaled and corrected using surface observations. Regional reanalyses, created by driving higher resolution models with climate data from global reanalyses, have resolutions of approximately 10–50 km [47,48].

### Projections of future climate

In this section, projections of future climate are discussed. Global climate models are briefly described, followed by the scenarios used to drive them.

#### Global climate models

Projections of future climate originate with global climate models (GCMs), which embody the current understanding of the dynamical, physical and biogeochemical processes that control the climate system [49]. The GCMs used for the fifth assessment report of the Intergovernmental Panel on Climate Change (IPCC), published in 2013, had horizontal resolutions between 60 and 150 km [50,51].

In addition to atmospheric processes, current GCMs include representations of the ocean and its circulation, aerosol particles, the land surface, land and sea ice, vegetation and the carbon cycle, and, more recently, atmospheric chemistry [50,51]. The ability of GCMs to simulate observed climate variables and their spatial patterns has continuously improved [51].

No two GCMs are identical; they contain different but plausible methods for representing climatic processes, numerical methods for solving equations and representations of processes which occur at spatial scales that cannot be resolved directly by the climate model [49]. There are two important consequences of the choices made when constructing climate models. First, a range of changes in temperature, rainfall and other climate variables are produced by different climate models when they are forced with the same estimates of future greenhouse gas emissions. Secondly, systematic errors (or “biases”) are apparent when comparing simulations of present day climate with observations. Correction of these biases is especially important when absolute thresholds are used, as in temperature-mortality models. Methods for correcting biases, often referred to as calibration, are discussed below.

#### Emissions scenarios

It is impossible to predict future emissions of greenhouse gas emissions and changes in land use. Projections of future climate are created by driving global climate models with greenhouse gas emissions or atmospheric concentrations prescribed in scenarios. Scenarios are neither forecasts nor predictions; they provide descriptions of possible future socioeconomic and technological changes, population growth and land use change, from which emissions of greenhouse gases can be estimated. The earliest scenarios used either a fixed increase in carbon dioxide levels (1% per year, for example) or involved executing a GCM with constant levels of carbon dioxide, at present day and doubled levels [50]. These simulations provided useful information on possible future climatic conditions, but no indication of when those conditions might occur.

The IPCC published a set of six scenarios (“IS92”) in the early 1990s [19] which were used to assess climate change for the IPCC second and third assessment reports that were published in 1996 and 2001. Increased understanding of the driving forces of emissions and assessment methodologies led to the production of a new set of scenarios which are described in the Special Report on Emissions Scenarios [20]. These “SRES” scenarios were derived from four different socioeconomic storylines based on various assumptions regarding population growth, technological changes, energy sources and land use [20]. None of these scenarios included policies to reduce greenhouse gas emissions. The SRES scenarios most commonly used are high emissions (A1FI, A2), medium emissions (A1B, B2) and low emissions (B1). Climate simulations using a subset of the SRES scenarios were used to inform the third and fourth assessment reports of the IPCC. About half of the studies in Tables 1 and 2 used climate data generated under one or more of the SRES scenarios.

A new set of scenarios based on radiative forcings were developed to replace the SRES scenarios; they were given the label representative concentration pathways, or RCPs [21]. The RCPs, unlike the SRES scenarios, are not based on socioeconomic storylines [50]. Instead, a specific emission scenario, including land use and land cover changes, was identified which would lead to each target radiative forcing trajectory [21]. The four RCPs include a mitigation scenario leading to a very low radiative forcing level (RCP2.6), two medium stabilisation scenarios (RCP4.5 and RCP6.0) and one very high emission scenario (RCP8.5). A comparison of global mean temperature changes and associated carbon dioxide levels shows that the SRES A1FI and RCP8.5 scenarios are similar, and the SRES A2 scenario lies between RCP6.0 and RCP8.5. SRES A1B is close to RCP6.0 and SRES B1 is similar to RCP4.5. The RCP2.6 scenario includes policies which result in net negative emissions of carbon dioxide, and so temperature changes projected with this scenario are notably lower than any projections using the SRES scenarios. Climate projections using four RCPs formed the basis of the Coupled Model Intercomparison Project 5 [52] (CMIP5). These projections were analysed extensively for the IPCC fifth assessment report [1,2,3]. Thirteen studies used climate data generated under one or more of the RCPs to estimate future mortality (Table 1).

Two studies used other scenarios. A study of future mortality in cities in the USA [22] used two different scenarios (“REF” and “POL3.7”), which are similar to the RCPs but have different radiative forcings. A study of future mortality in the Netherlands [53] used four scenarios (“KNMI’14”) which were based on a downscaled subset of the CMIP5 projections.

No likelihood is attached to the IS92, SRES or RCP emissions scenarios. They are assumed to be equally plausible representations of future emissions. Ideally, future climates generated under all scenarios within a group (i.e., all SRES or all RCPs) would be used to explore the impacts of different policy options on projected mortality. However, it may not be practical or possible to do so owing to the large volumes of data involved. Some downscaled climate model datasets were created using a single emissions scenario (see next section).

### Downscaling climate simulations

GCMs depict the climate using a three dimensional grid over the globe. Current GCMs have resolutions between about 60 km and 150 km [51]. For many impacts studies, climate data are required at higher spatial scales, so downscaling is required. Downscaling refers to a process whereby climate information at large spatial scales is used to create projections at smaller spatial scales. There are two main approaches for downscaling, dynamical and statistical.

Dynamical downscaling involves the use of a regional climate model (RCM) over a smaller domain (e.g., a continent or country) at higher horizontal resolution. RCMs, like their parent GCMs, are based on physical principles. The resolution of RCMs has also increased over time. The RCMs used in earlier studies had resolutions of the order of 50 km (Table 1), whereas some more recent RCM simulations have resolutions between 10 km and 25 km. Occasionally, RCMs with higher horizontal resolutions have been used (250 m [54], 1 km [55], 4 km [31]).

Statistical downscaling is based on relationships between local climate variables, such as temperature and rainfall and large-scale “predictor” variables such as air pressure or temperature. First, these relationships are derived using observations of climate variables. Next, these relationships are applied to projections from GCMs to produce local climate data for the future. Statistical methods have been used to produce monthly, daily and sub-daily climate data at local scales [23,56,57].

Three studies used both downscaling methods [34,35,43]. A regional climate model was used to dynamically downscale global climate model simulations to a higher resolution, and then statistical methods were used to produce data at either specific locations or on a regular grid. A similar combination of methods was used to create the KNMI’14 scenarios [53].

Dynamical and statistical downscaling methods have advantages and disadvantages. RCMs can produce a wide range of climate variables at high spatial and temporal resolutions, but are computationally expensive to execute. Some ensembles of regional climate model projections have been created using only a single emissions scenario. For example, the ENSEMBLES project [58] used the SRES A1B scenario, whereas the North American Regional Climate Change Assessment Program [59] (NARCCAP) used the A2 scenario. The CORDEX initiative [60] has produced downscaled climate data using multiple global and regional climate models under the RCP scenarios for many land regions of the world. Climate data from RCMs are likely to require calibration, as any errors in the driving GCM climatology will also be present in the RCM climate. Calibration of climate data is discussed below.

Statistical methods require less computational resources than RCMs, but need a long series of observations of the climate variables of interest in order to establish robust relationships with large scale predictors. The relationships can vary temporally and spatially. Additionally, statistical methods implicitly assume that the relationship between local and large scale variables does not change over time, which may not be true. Statistical downscaling methods generally incorporate a calibration step.

Four studies [43,61,62,63] used a weather generator to create daily series of climate variables for specific locations. A weather generator is not, strictly speaking, a downscaling method, but can be used with other downscaling techniques to produce local climate information. A weather generator is a statistical model designed to generate synthetic but realistic series of climate variables of an arbitrary length. Weather generators incorporate a stochastic rainfall model which simulates rainfall sequences. Other climate variables (such as daily maximum and minimum temperatures) are then calculated from regression relationships with the rainfall amounts and current state (i.e., wet or dry) [61]. Most weather generators operate on daily time scales, although some also produce hourly values of climate variables [64]. Data for future time periods can be created by either adding climate change factors to a present-day series, or modifying the relationships between the weather variables. A long standing issue with weather generators is their inability to reproduce long periods of persistent weather such as warm temperatures and droughts [64].

### Climate variables

The very first study of future heat-related mortality used prescribed temperature increases which were based on a single GCM simulation [28]. Six other studies also used prescribed increases in temperature [32,33,65,66,67,68]. Of these studies, two [33,65] added temperature changes based on global mean changes to observed daily summertime temperatures of the cities under study. Analyses of regional temperature changes projected by global models shows that simulated temperature changes over individual land points are almost always greater than global mean changes [2,3]. The exact change in temperature varies considerably within a given region [2]. Modelled temperature changes for the location of interest should therefore be used.

Most studies have used climate data from the grid box that encloses the location of interest. Several studies [37,69,70,71,72] interpolated modelled data to the point(s) of interest, whereas two others [73,74] used the average of the values from the central and eight surrounding boxes.

Several different climate variables have been used to project future mortality (Table 2), although justification for the choice of variable is rarely given. Two studies [32,75] related monthly mortality to monthly means of daily temperatures. Most other studies used daily data, of which the most common variables were daily maximum and daily mean temperatures (Table 2). Two studies used daily minimum temperatures [31,51], and so relate mortality to hot nights instead of hot days.

Some studies modelled mortality using variables calculated from temperature and humidity which are thought to be physiologically relevant (S1 Appendix). Apparent temperature, a function of air and dew point temperature was used by a number of studies [30,40,46,65,76,77,78]. One study [79] used the Humidex and another [31] the heat index [80]. A study of mortality in Vienna [73] used physiological equivalent temperature (PET), which is calculated with a heat balance model of the human body.

Many of the climate variables (daily temperatures, apparent temperatures, etc) used to construct mortality models are correlated [81], so that the choice of variable may not be important. However, another study compared the numbers of days identified as being important for heat-related deaths using four different heat-health warning systems based on different climate variables [82]. The numbers of hot days were dependent on the variable chosen, even though some of the variables were highly correlated. A study of mortality in seven US cities found that the correlations between different variables (daily minimum, mean and maximum temperatures) were weaker for the extremes than for the entire distribution [83]. Additionally, daily maximum or mean temperatures were more strongly associated with mortality than minimum temperatures [83]. Some climate models project larger increases in daily maximum temperatures during the warm season than daily mean or minimum temperatures [84]. The use of mortality models based on daily minimum or mean temperatures may therefore produce lower estimates of future mortality than models based on daily maximum temperatures.

A small number of studies [44,56,67,76,85,86,87] classified air masses into different weather types based on temperature, humidity and other characteristics. Models linking mortality with metrics such as apparent temperature (Table 2; S1 Appendix) were then built separately for oppressive weather types (those associated with high temperatures and/or high humidity likely to cause large increases in mortality) and other weather types. This approach has the advantage of not requiring any downscaling of global climate model data to local levels. However, the ability of climate models to simulate the correct numbers and seasonality of the air mass types was not always assessed. Errors in the modelled circulation could result in over- or under-estimation of oppressive air mass types which would impact upon projected changes in mortality.

### Calibration of climate data

Climate models have improved considerably since they were first developed in the late 1960s [51]. Despite the continuous developments, systematic errors or biases (for example, over- or underestimation of summer temperatures, simulation of too many wet days) are apparent when simulations of present day climate are compared with observations, although the magnitudes of these biases have steadily decreased [51]. Correction of biases in the modelled climate data (a procedure referred to as calibration in the present study) is therefore required. Any biases in the modelled climate would affect the estimated baseline mortality and projected changes in mortality, which can be significant [42,88]. Despite this issue, four studies appear to have used raw (i.e. uncalibrated) climate model data to estimate future mortality [63,71,77,89] which would introduce errors into their estimates.

Almost all of the studies in Table 1 have combined data from climate models with local observations of climate to reduce biases in the modelled data. Regardless of the method used, the climate change information from a GCM or RCM is often at a coarser resolution than the local climate data. Hence, the local scale characteristics of the calibrated data are dependent on the observations whereas climate change effects are controlled by coarse-scale data from climate models [90].

The calibration methods used by the studies are summarised in Table 2 and most fall into one of two groups, “delta” and “shift”. Under the “delta” method, differences in modelled climate between a baseline and future period (called change factors) are calculated and added to an observed time series [27]. In many cases, the time scales of the change factors were different to the observed time series. Monthly or annual mean changes in temperature were added to observed daily temperatures in most studies (Table 2). One study calculated monthly temperature changes from climate model simulations and added them to observed monthly mean temperatures [75]. Two studies calculated average daily temperature changes and added them to observed daily temperatures [51,91].

Seven studies used the shift method to calibrate their climate data (Table 2). Modelled and observed data over a common period were used to calculate daily or monthly correction factors which were then added to the modelled data over all time periods [27]. Seven other studies used the percentile approach to calibrate their modelled data (Table 2). The percentile of the threshold temperature from observations (above which excess mortality occurs) is applied to modelled temperatures in the baseline period. The new threshold is then used with the modelled data. The percentile approach is equivalent to using the shift method with a single value, as the same value would effectively be added to the entire modelled temperature distribution.

Other methods have been used to calibrate climate data, including quantile mapping and fitting of functions to the distributions of the data. Four studies used quantile mapping methods [37,40,62,92]. Empirical cumulative distribution functions of the modelled and observed data are used to calculate a correction factor for each pair of data values. This method can be extended to include modelled changes between a baseline and future periods.

Two studies fitted logistic distribution functions to the distributions of the modelled and observed temperatures [42,88]. The logistic function is defined by two parameters analogous to the mean and standard deviation of a normal distribution. Changes in the parameters between the functions fitted to the modelled future and baseline temperature distributions were then added to the respective parameters estimated from the observational distribution. Finally, the resulting distribution was sampled to produce a daily series of calibrated temperatures for the future periods.

The ability of various calibration methods, including the “delta”, “shift” and quantile mapping, to reduce errors and reproduce high and low extremes of temperature distributions has been assessed [27]. It was found that the delta and shift methods performed the worst in reproducing the higher and lower temperatures in the distribution compared with quantile mapping. The delta method performed the worst overall, whereas the shift was the worst method for reproducing temperatures in the upper half of the distribution [27]. The shift method implicitly assumes that the biases are stationary, so that the correction factors calculated for the present day are applicable to future periods. This assumption of constant biases may not be correct, and could be invalidated seasonally, geographically, and also with the amount of global warming [93].

### Heat waves

Most of the studies reviewed have considered the well established effect of general summertime temperatures on mortality [7]. Twelve of the studies considered the effects of heat waves on future mortality and are summarised in Table 3. Heat waves (a period of consecutive anomalously hot days and/or hot nights) are comparatively rare events, whereas warm and hot days occur in most if not all summers. Hence, mortality attributable to hot days is generally much larger than mortality from heat waves. A wide range of heat wave definitions have been used (Table 3), which makes comparisons between studies difficult. Five studies modelled mortality as a function of the lengths of the heat waves [31,35,40,94,95], whereas five others used temperatures during the heat waves [29,55,70,92,96]. One study calculated a separate mortality risk for each day in sequence of the heat wave [79], and another modelled mortality as a function of the number of heat wave days in the hot season [37].

**Table 3:** Studies which explicitly calculated mortality from heat waves.

Study and Reference	Location	Variable	Heat wave definition(s): Threshold(s), Months, Years	Minimum length(s) / days	Mortality depends on
Heaviside et al. (2016b) [55]	West Midlands (UK)	TM	A heat wave in UK, 1–10 August 2003.	10	TM
Roldán et al. (2016) [96]	Zaragoza (Spain)	TX	38°C (99^th^ percentile of TX)	1	TX
Kim et al. (2016)[35]	South Korea	TX	33°C	1	Square of length
Murari et al. (2015) [37]	India	TX TX	a) TX > 45°C b) TX > average of 1970–1999, March-May + 7°C and TX > 40°C.	1 1	Heat wave days per season
Zacharias et al. (2015)[95]	Germany	TM	TM > 97.5^th^ percentile	3	TM, Length
Wu et al. (2014)[31]	Eastern USA	HI TM TX TN	a) HImin > 26.7°C and HImax > 40.5°C. b) TM > 95^th^ percentile c) T1 = 97.5^th^, T2 = 81^st^ percentile‡ d) TN > 95^th^ percentile N.B. (b)–(d) based on temperatures from May-September, 2001–2004	1 2 3 2	Length
Hajat et al. (2014)[29]	UK	TM	TM > 98^th^ percentile 1993–2006	3	TM
Zhou et al. (2012)[92]	Three cities in Alabama	TX	TX > 90^th^, 95^th^, 97.5^th^, 99^th^ percentiles 1991–2000	2	TX
Ostro et al. (2012)[70]	Four cities in Spain	TM	TM > 95^th^ percentile 16 May– 15 Oct 1960–1990	2	TM
Peng et al. (2011)[94]	Chicago	TX	T1 = 97.5^th^, T2 = 81^st^ percentile‡ May–October 1981–2000	3	Length
Jackson et al. (2010)[79]	Washington State	HX	HX > 99^th^ percentile 1970–2006	1	Day in sequence
Hayhoe et al. (2004)[40]	Los Angeles	AT	AT > 34°C	3	AT and length

*Variables–*TX, TM, TN are daily maximum, daily mean and daily minimum temperatures respectively. AT is daily apparent temperature (section S2.1), HI is the heat index [80] and HX is the Humidex [79]. *Heat wave definitions–*the threshold(s) used with the period of data (a range of years) and (where applicable) the months. For example, 95^th^ May-Sep 1961–1990 would mean the threshold was defined as the 95^th^ percentile of daily temperatures over the period 1961–1990 using data from the months of May to September only. If no month range is given, the threshold was calculated using temperatures from all months. *Minimum length–*the minimum number of consecutive days classed as a heat wave. *Mortality depends on–*the variable used to calculate mortality; length refers to the number of days in the heat wave.

‡This definition uses two thresholds (T1 and T2) of daily maximum temperatures (TX). A heat wave is defined as a period when (a) TX > T1 for at least 3 days, (b) the average of TX over the heat wave is greater than T1, and (c) TX > T2 for every day during the heat wave.

Only three studies calculated mortality from both individual hot days and heat waves. The importance of heat waves differed considerably. A study of future mortality from hot days and heat waves in three cities in Alabama found that deaths from heat waves were at most a few percent of the deaths from high temperatures [92]. In contrast, deaths from heat waves were estimated to be of similar magnitude to deaths from individual hot days for four cities in Spain [70]. In a study of heat-related mortality in UK regions during the twenty-first century, the extra mortality from heat waves was only important for London [29].

A systematic review of heat wave definitions and associated mortality [97] concluded that the impact of heat waves on mortality was important, but the magnitude of the effect varied under different heat wave definitions. Generally, the higher the temperature threshold used, the higher the impact on mortality. The intensity of the heat wave appeared to be more important for mortality than the duration. However, it was unclear whether the effects of the intensity and duration of heat waves were independent or interactive [97].

### Adaptation

There is considerable evidence to show that populations in some areas have adapted to warmer temperatures over past few decades [98,99,100,101,102,103]. Adaptation can occur via physiological acclimatisation and behavioural changes. Other mechanisms include improved health care, provision of heat-health warning systems [104] and increased installation of air conditioning systems [46]. Adaptation to warmer temperatures can occur within a season and over many years. Only two studies included within-season adaptation [40,85], where modelled mortality at the end of the warm season would be lower than at the beginning given the same climatic conditions.

Most of the methods by which longer-term adaptation in its various forms has been included in quantitative estimates of future mortality are simplistic. In many studies, the mortality threshold was increased by an arbitrary amount with no justification or reference to epidemiological evidence (Table 1). Some studies reduced the gradient of the temperature-mortality relationship [30,46,53,75,85,105]. Other studies estimated the effects of adaptation by extrapolating mortality-temperature trends into the future [73], or simply ignoring mortality in the first few days of a heat wave [44]. A small number used the “analogue city” approach, where the mortality model for a city with a warmer climate is applied to the city under study [28,69,86]. None of the studies have considered “negative adaptation”, where communities become less well adapted to warmer temperatures, owing to failure of power generation or transmission grids in populations accustomed to using air-conditioning, for example [106].

### Population changes

Future changes in population and demographics (specifically aging) are important when calculating heat-related mortality. The numbers of deaths would be expected to increase owing to larger populations and projected higher proportions of older people who would be more vulnerable to the effects of high temperatures. Many studies did not include estimates of population growth in their projections (Table 1) and so would underestimate the numbers of heat-related deaths in the future. Several studies have shown that projected numbers of deaths were considerably higher when population growth and changes in demographics were included, compared with results using a static population [29,55,70].

## Discussion

Sixty three papers estimating future mortality from high temperatures and heat waves have been reviewed. These studies used a wide range of surface observations and climate model projections (Tables 1 and 2). All the studies indicate that heat-related mortality would increase under a warming climate. The projected impacts of climate change on mortality are highly dependent on the future scenarios and climate models chosen. The majority of studies have used a small number of climate simulations without considering where they lie within the range of future projections. The difficulty in obtaining and processing data from climate models is likely to have been one factor in older studies, although access to climate model data has greatly improved in recent years. The use of climate information from a small number of models means future mortality estimates could be biased low or high. Ideally, all available simulations would be used to estimate future mortality. Alternatively, a subset of the climate models could be selected which captures key regional climate processes and the range of possible changes in climate [18,53]. Calibration of data from climate models is required to reduce the impacts of any biases. It should be noted that no calibration method will remove all deficiencies in the modelled climate, such as over- or under-prediction of the persistence of periods of hot and cold weather.

Uncertainty in estimates of future mortality originates from several sources, including climate models and emissions scenarios [88], the calibration method, the mortality model [70], treatment of adaptation [44,46] and future population changes [29]. Consequently, estimates of future mortality for the same city can vary considerably between different studies. As an example, mortality estimates for Chicago from five studies are compared in S3 Fig. These estimates vary by a factor 4 or more. In areas where surface observations are sparse and the terrain is complex, uncertainty in interpolated or gridded data derived from observations can be large [90]. This issue has not been considered in projections of climate impacts on health.

The relative importance of these various sources of uncertainty is likely to change temporally. In the near future, the choice of mortality model might be one of the larger sources of uncertainty [70,84], whereas over longer time periods climate model uncertainty, the choice of scenario and treatment of adaptation would become more important [40,44,70,73,75,91,107,108]. A study of mortality in 14 European cities for the end of the twenty-first century using six different adaptation methods showed that the uncertainty originating from the adaptation methods was mostly larger than uncertainty from climate models and emissions scenarios [46].

Two studies [27,78] used median or mean changes in temperature from an ensemble of climate model simulations and did not consider the range of projections. Two others [109,110] estimated mortality using projections from multiple climate models but only reported median changes in mortality. There were large differences in projected mortality in all of the studies which used data from two or more climate models driven by same emissions scenario (e.g., [76,88,111,112]).

A few studies quoted future mortality estimates as averages or ranges across different emissions scenarios [31,62,84,107]. Another study appears to have used a weighted average of projections from two scenarios which were created with two different models to estimate future mortality [79]. These mortality averages and ranges are very dependent on the scenarios and models used and are therefore potentially misleading. Results should be presented separately for each scenario.

About half of the studies reviewed here used climate data generated with medium or high emissions scenarios (SRES B2, A1B, A1FI and A2; RCP6.0 and RCP8.5). A smaller number used lower emission scenarios (SRES B1; RCP4.5). The Paris Agreement to limit global warming to less than 2°C and pursue efforts to limit the temperature increase to 1.5°C was ratified in November 2016. The RCP2.6 scenario is the only one consistent with the aims of the Paris Agreement, but just three of the studies [36,37,46] considered it. Further studies of future mortality using the RCP2.6 scenario are therefore required. However, even if global warming was limited to 2°C, increases in numbers of hot days and lengths and intensities of heat waves are still likely [113].

The use of short time lengths for a baseline and future periods (for example, 3–5 years; [31,33,51,69,77]) should be avoided. Regional climate in most areas of the world is highly variable, and the climate in a short period could be anomalously warm or cold relative to a longer-term average. Very different projections of mortality could be obtained if different years had been chosen for the baseline and future periods. One study [30] showed that projected mortality using 3-year and 30-year future periods were very different, by about a factor of 2.

Some studies have only considered mortality in the summer months or warm season (for example, June–August or May–September in the Northern Hemisphere). This approach would exclude unusually warm months outside of these periods and bias mortality estimates low. For example, in 2003 and 2011, temperatures in April in London exceeded the mortality threshold used in [29] for several days. The length of the warm season would be expected to expand in the future [44], increasing the chance that some warm days and the associated mortality would not be included if a fixed time period was used.

The variable chosen to model heat-related mortality can also affect estimates of future mortality. A study of mortality in 107 cities in the USA [81] used several different climate variables (such as daily maximum temperature and apparent temperature). The best variable for modelling mortality varied between the different cities. Another study [78] estimated future mortality in Europe using two different variables and obtained similar (but not identical) results. In contrast, a study of mortality in the UK [114] found that mortality was best modelled using daily maximum temperatures. Some climate models project larger increases in daily maximum temperatures during the warm season than daily means and minimums [84]. Estimates of future mortality will be partly dependent on the variable chosen, but the importance of variable choice is likely to vary with location.

The urban population is growing and is expected to continue increasing in the future [115]. Urban areas have their own climates which are different to surrounding rural areas. They are generally warmer than rural areas, especially at night, owing to absorption and release of heat by buildings, waste heat from energy use, and a lack of surface moisture [55,116]. The temperature differences between towns and cities and rural areas are referred to as the urban heat island (UHI). The UHI can reach values of up to 10°C in large cities [55]. Urban populations are therefore exposed to higher temperatures than rural populations. Urban temperatures in the future could increase from expansion of urban centres as well as the warming climate [116].

Many climate models do not simulate urban climates, so that future heat-related mortality within cities is likely to be underestimated. Three of the studies in Tables 1 and 2 used models which explicitly simulated urban climates [54,55,61]. One study [61] used a modified weather generator to simulate the climate of London. The effects of climate change as well as increased urbanisation and anthropogenic heat emissions on mortality in London were examined. Future mortality increased as a result of climate change, and the inclusion of increased urban land use and anthropogenic heat release resulted in a further increase in mortality of about 10–15%.

Another study calculated the effect of the urban climate on mortality in a large city in the UK during a severe heat wave [55]. Calculations of heat-related mortality over the same period in which urban areas had been replaced by a rural land type were about 50% smaller. Future mortality, estimated by adding temperature changes from a regional climate model to the modelled present-day temperatures, was notably higher when urban temperatures were used compared with the rural values.

The impacts of increased urbanisation on the climate of a city in a semi-arid area have been studied [116]. Daily minimum temperatures were increased by a larger amount than daily mean temperatures under greater urbanisation; little effect was simulated on daily maximum temperatures. Changes in heat-related mortality based on daily minimum temperatures were notably higher than estimates based on daily mean temperatures [116]. Changes in mortality based on changes in daily maximum temperatures were small and negative, so that the increased urbanisation acted to reduce mortality slightly. Similar changes in temperatures and impacts were found in a study of heat stress (but not mortality) in Sydney resulting from urban expansion and climate change [117].

These studies mates highlight the fact that changes in urban climates are different to those in rural areas. The extra heat from anthropogenic activities further raises urban temperatures but was only considered in one study [61]. Many global and regional climate models do not explicitly simulate urban climates. High resolution model simulations of urban areas are few in number, probably due to the high computational cost of running such models. If climate data from these high resolution climate simulations are used for estimating future mortality, the variable used to model mortality needs to be chosen with care.

One issue with heat-mortality models occurs when they are used with temperatures higher than those used to construct them. Projections based on simple linear models could underestimate mortality, especially when extreme temperatures are experienced. Some non-linear models have very steep gradients for high temperatures [42,70,78]. A small increase in temperature would produce a very large increase in mortality which might be unrealistic. It could be insightful to compare the projected temperature changes with the calibration range to understand how much extrapolation is occurring.

When presenting estimates of future mortality, results using no change in population and demographics should be given alongside results with population and demographic changes. The separate effects of changes in climate and changes in population on mortality can then clearly be seen. For example, in a study of heat-related mortality in the UK [29], mortality was estimated to increase by 66%, 257% and 535% by the 2020s, 2050s and 2080s respectively when population size and aging was included, but only by 46%, 169% and 329% if population size and ages were held constant. Large differences in projected mortality were calculated in other studies using different scenarios of population growth and aging [70,118].

Adaptation or acclimatisation of the population to warmer temperatures is important when estimating future mortality [119]. There is evidence from the epidemiological literature that in some locations, heat related mortality has decreased over time [98,99,100,101,102,103]. These results might suggest that existing measures are keeping pace with warming so far, but it is unclear if and how such measures could continue to succeed in the future. Some degree of adaptation to heat is likely to have occurred naturally [98]. Evidence of short-term adaptation to heat is also supported by physiological studies [120]. It is not possible to say how much of the decreased sensitivity to heat demonstrated in these studies is due to physiological, behavioural or adaptive structural changes in the environment (e.g., increased availability of air conditioning, planting of trees to provide shade, etc).

In brief, there are two aspects which can be considered: adaptation within the warm season and longer term adaptation to warmer temperatures. Two studies accounted for possible in-season adaptation, so that mortality at the end of a warm season would be lower than at the beginning, given the same climatic conditions [40,65]. Longer term adaptation has been estimated using several different methods. Three studies used the differences in mortality between warm and cool summers [40,85,107]. Many other studies have estimated the possible effects of adaptation by increasing the mortality threshold temperature by a fixed amount, typically between 0.5°C and 4.0°C (e.g., [42,46,61]). Other studies changed the slope of the exposure-response function [30,46,53,75,85,105]. These changes to the threshold temperatures or slopes are often arbitrary and are rarely supported by epidemiological evidence [46].

The differences in future mortality estimates incorporating six different adaptation models has been studied for selected European cities [46]. This study showed that uncertainty in future heat-related mortality resulting from different adaptation methods was larger than uncertainty associated with emissions scenarios and climate models. A study of mortality in Beijing reached similar conclusions [105]. There is a need for greater evaluation of intervention methods to improve modelling of adaptation within epidemiological models [46].

Heat waves are rare events, meaning there are few examples to study. It is therefore difficult to assess by how much mortality could be elevated by the persistence of the hot conditions during heat waves. The effects of heat waves on future mortality have only been assessed by twelve studies (Table 3), and of those only three modelled mortality from both hot days and heat waves. The importance of heat waves for excess mortality varied considerably. Heat waves are projected to become more frequent, hotter and longer as the climate warms [1]. Whether the importance of heat waves compared with individual hot days for mortality would also increase in the future is unclear.

The timing of hot days and heat waves may also be important. Those that occurred early in the warm season in temperate zones might have a larger effect on mortality than those which happened later [7,85,121,122]. High temperatures have the largest effect on older people, whereas prolonged heat waves can impact on the entire population. A modified mortality relationship may be needed for heat waves than for the general effect of high temperatures. It is unclear whether the lengths and intensities of heat waves act independently or synergistically on mortality [97]. Further research is needed to fully understand the effects of heat waves on mortality.

### Factors that have not generally been considered

There are several other factors which could be important when estimating future heat-related mortality, but have not been included in the studies reviewed here. Summer mortality from high temperatures may be moderated by mortality in the previous winter [123,124,125]. If mortality during winter was low, mortality in the following summer could be elevated, owing to a larger number of vulnerable people. Similarly, a winter with high mortality could mean mortality in the following summer would be reduced. However, aside from a few studies, the epidemiological evidence for linkages between winter and summer mortality are not well established. Any linkage may reduce in importance as the climate warms.

The importance of the socioeconomic status of the population when calculating future mortality is unclear. Two studies [126,127] found some evidence to show that populations in deprived areas of Chicago and Paris respectively were more vulnerable to heat related mortality than those in affluent areas. In contrast, a study of mortality in Australian cities [87] saw little or no evidence for modification of the temperature-mortality relationships by socioeconomic status.

A rapid change in temperature within a day (the diurnal temperature range, DTR) could be a risk to human health [128]. Those with cardiovascular and respiratory diseases are most at risk from large changes in DTR. The elderly and children appear to be more susceptible to large changes in the DTR than other age groups. Further research is needed to confirm and understand any effects of DTR on health and mortality [128]. So far, DTR has not been included as an explanatory factor in models used to estimate future mortality.

### Strengths and limitations

This review is the first to synthesise and critically assess the use of information from climate models to estimate future heat-related mortality. Additionally, three factors which have not been considered so far in projections of future mortality were identified.

There are several limitations of the current review. Many different combinations of keywords were used to identify relevant articles, but some may have been missed. Only articles published in English were searched for, so any relevant studies in other languages will have been omitted. The databases searched did not index journals in fields such as economics and social sciences. Two relevant articles published in economics journals were found in reference lists of other papers, and there may be other papers in similar journals which would be of interest. Some studies may not have been published, especially if they had negative results, so some degree of publication bias cannot be ruled out. All articles in which an estimate of future heat-related mortality was reported were included regardless of the quality of the study. The assessment of quality is subjective, and was not included in the eligibility criteria. However, inclusion of those papers regarded as low quality would not alter the conclusions or recommendations of this review.

## Conclusions

Heat-related mortality is now recognised as a serious issue which is likely to increase in severity as the climate warms. Studies of future mortality would benefit from more interdisciplinary collaborations to improve the quality of research and results and also to broaden the interest and readership of work that is likely to be important for public policy across a number of sectors. There are very few studies of the effects of warm temperatures on mortality outside of high-income countries. More studies are needed in middle and low income countries, and in sub-tropical and tropical areas. Additionally, there are very few studies specifically estimating future mortality in rural populations.

Adaptation will play a key role in reducing the effects of a warmer climate on heat-related mortality. In some locations, heat-related mortality has fallen over the past few decades, suggesting existing measures are keeping pace with the rate of warming. It is unclear whether these existing measures will continue to succeed in the future; some newer measures are likely to be needed. Methods by which adaptation has been included in mortality models are often simplistic and are not linked to epidemiological evidence. More research is needed to improve the representation of adaptation within mortality models.

Uncertainty in projections of future mortality originates from several sources, but only a small number of studies have partially or fully addressed this issue. In particular, uncertainty from the choice of climate model simulation(s) is not often considered. Ideally, climate projections from multiple models under different emissions scenarios with a range of greenhouse gas emissions would be used to estimate future heat-related mortality. Alternatively, a subset of the climate model projections which captures the range of climate change over the area of interest could be selected.

Estimates of future heat-related mortality are partially controlled by two competing effects: population growth and aging would act to increase mortality, whereas adaptation would reduce mortality. It would be of interest to understand how the magnitudes of these two effects change temporally within epidemiological models and with the amount of warming.

The recognition of and interest in heat-related mortality may provide opportunities for more interdisciplinary studies involving both epidemiologists and climate scientists to better estimate the impacts of high temperatures on health and properly include uncertainty in the projections of future climate. The outcomes of such studies should be directed toward answering policy-relevant questions and contributing toward the design of suitable adaptation measures.

## Recommendations

There are many different factors to be considered when estimating future heat-related mortality. Much depends on what data are available for any given area and the purpose of the research, for example, exploring worst case scenarios and impacts of different policy options. One important recommendation from this review is transparency in reporting, ensuring the data used fit the purpose of each study and any limitations are reported.

An example ‘checklist’ for authors to consider when publishing results using climate model projections is suggested in Table 4.

**Table 4:** Suggested checklist for studies using climate model projections.

Area that quality criteria pertain to.	Example quality appraisal question	Has this item been reported in the study?
Global climate models	Has the uncertainty arising from GCM outputs been taken into account when reporting results?	
Emissions scenarios	Have the emissions scenarios used been well justified and do they fit the purpose of the research? (e.g. do the models include scenarios which cover all plausible policy options)	
	Where different emissions scenarios have been used, have the results been presented with transparent justification for their selection and is it clear where they lie within the range of projections?	
Downscaling climate simulations	Have the models used for projections been downscaled using a recognised method?	
Climate variables	Has the study used climate data for the local area of interest?	
Have the climate data been calibrated?	Which are the best methods for calibrating climate data? Or, just that climate data should have been calibrated.	
Epidemiological Models	Are there sufficient data to establish the baseline mortality? Have potential confounders (e.g., air pollutants) been considered?	
Population changes, including aging	Have future population numbers been estimated and aging taken into consideration?	
Adaptation	Has adaptation of the population to warmer temperatures been considered? If so, is the method related to epidemiological evidence?	
Results	Show results with/without population changes and adaptation. Ensure results can be converted to alternative units to aid comparison with other studies (e.g. between deaths per 100,000 population and total deaths)	

## Supporting information

S1 Checklist PRISMA 2009 Checklist. (DOCX)

S1 Fig Cities for which future mortality has been estimated. The symbols indicate the number of studies of mortality for that city.
(PDF)

S2 Fig Time periods considered in each study of future mortality. Magenta lines indicate the periods of observations used. Green and black lines show the model baseline and future time periods. Dashed lines and open symbols indicate time periods implied but not stated by the authors, or where prescribed temperature increases are assumed to represent the indicated time period. Single years are shown by solid or open circles.
(PDF)

S3 Fig Present day and estimated future mortality rates for Chicago. Mortality rates are in units of deaths per 100,000 of population. Mortality shown in magenta and green were calculated using high (SRES A1FI, A2) and low (SRES B1) emissions scenarios respectively. Mortality rates in grey were estimated using other scenarios. Error bars (where shown) represent the lowest and highest estimates using data from multiple climate models with the same mortality model. The estimates from Kalkstein and Greene (1997) were made using three different GCMs, and assume full adaptation of the population to the future temperatures. The mortality estimates for Chicago were normalised to deaths per 100,000 of population using census data for the specified year: Kalkstein and Smoyer (1993) - 1970 census, population 3366957. Kalkstein and Greene (1997) - 1980 census, population 3005072. Greene et al. (2011) - 2000 census, population 2896000. Petkova et al. (2014) - 2010 census, population 2707120. Hayhoe et al. (2011) quoted mortality as deaths per 100,000 and so their results are shown without any modification.
(PDF)

S1 Appendix Calculation of metrics which combine temperature and humidity. (PDF)
