# Supplementary figures and images for: The use of climate information to estimate future mortality from high ambient temperature: A systematic literature review
Source: PLoS One. 2017 Jul 7;12(7):e0180369. doi: 10.1371/journal.pone.0180369 (PMC5501532; doi:10.1371/journal.pone.0180369)

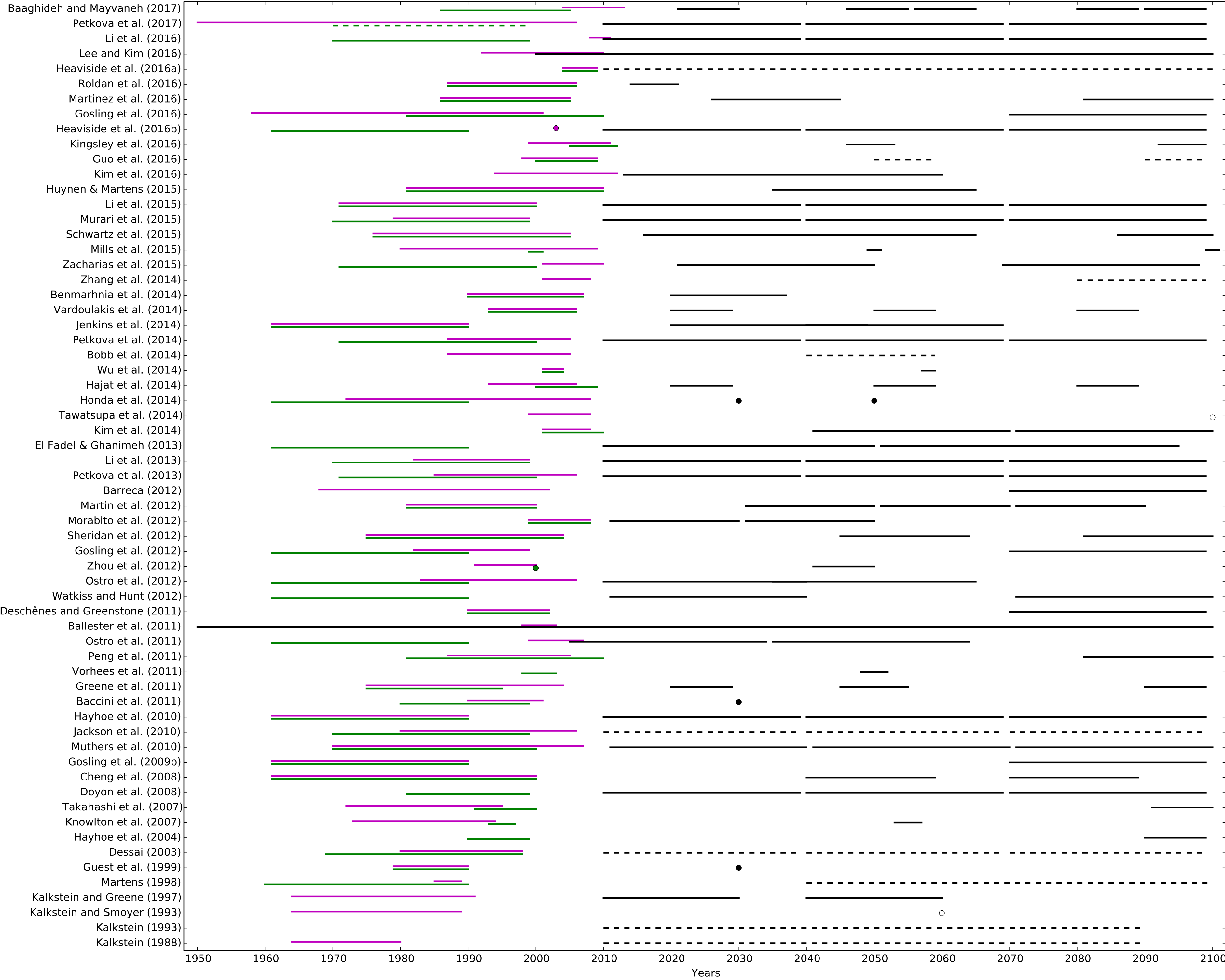

Supplement: S1 Fig — The symbols indicate the number of studies of mortality for that city. (PDF) [file pone.0180369.s002.pdf]

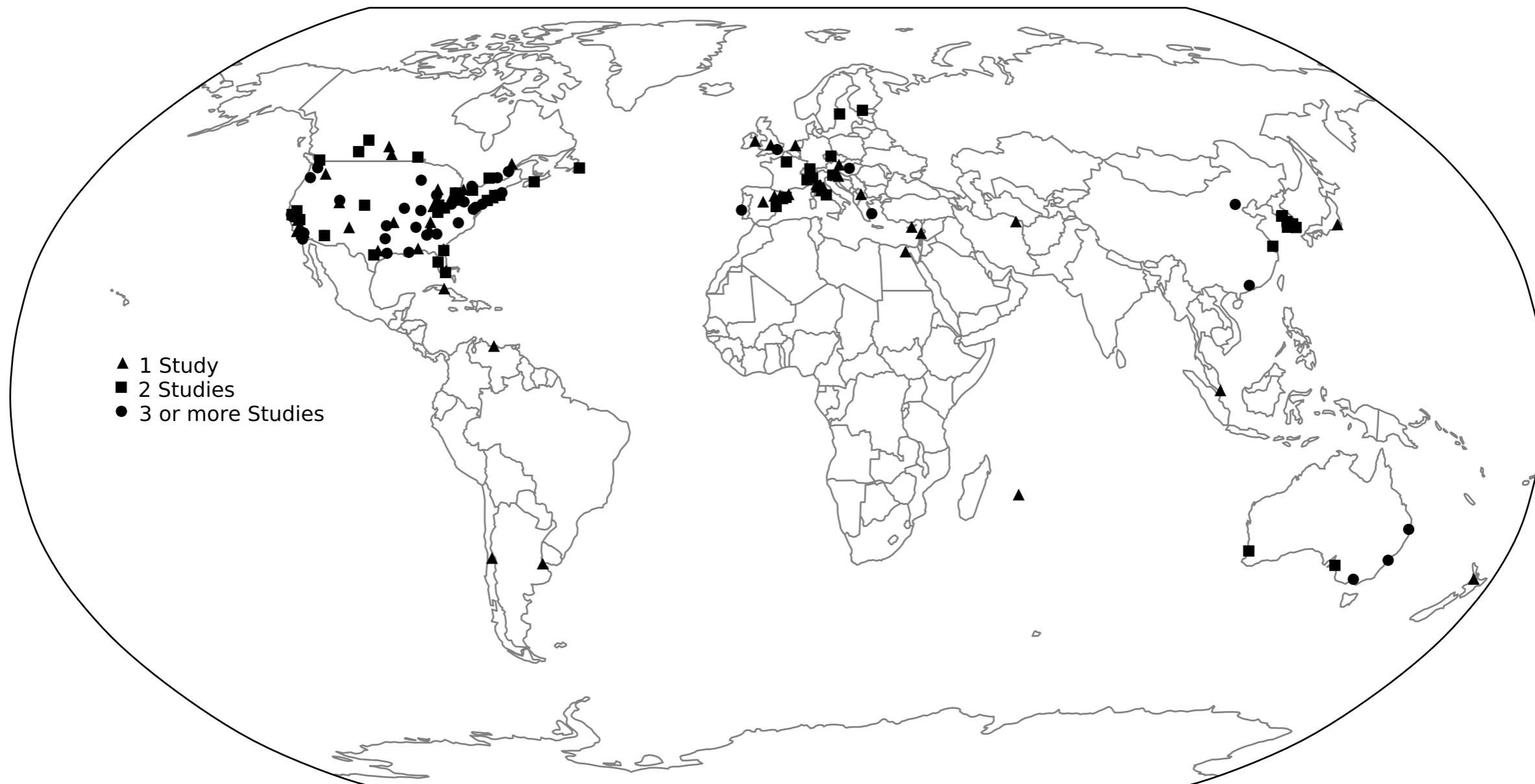

Supplement: S2 Fig — Magenta lines indicate the periods of observations used. Green and black lines show the model baseline and future time periods. Dashed lines and open symbols indicate time periods implied but not stated by the authors, or where prescribed temperature increases are assumed to represent the indicated time period. Single years are shown by solid or open circles. (PDF) [file pone.0180369.s003.pdf]

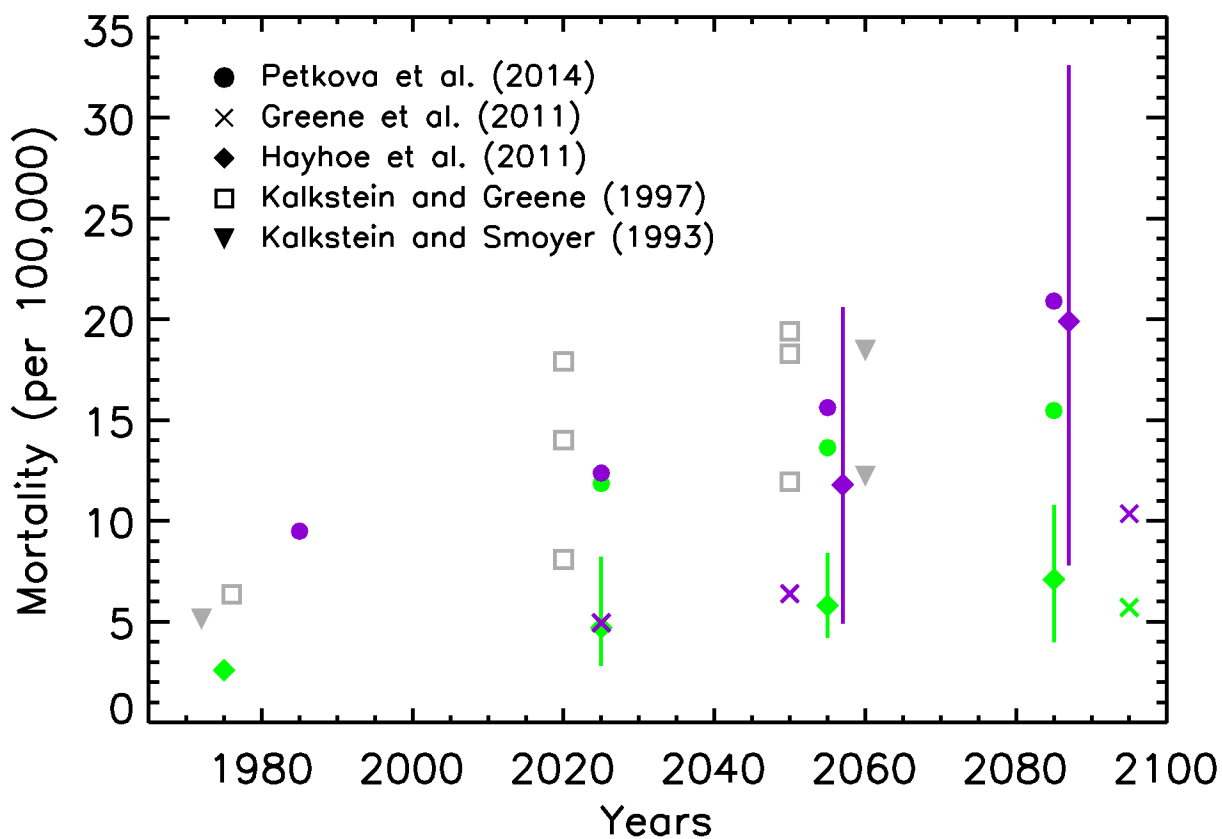

Supplement: S3 Fig — Mortality rates are in units of deaths per 100,000 of population. Mortality shown in magenta and green were calculated using high (SRES A1FI, A2) and low (SRES B1) emissions scenarios respectively. Mortality rates in grey were estimated using other scenarios. Error bars (where shown) represent the lowest and highest estimates using data from multiple climate models with the same mortality model. The estimates from Kalkstein and Greene (1997) were made using three different GCMs, and assume full adaptation of the population to the future temperatures. The mortality estimates for Chicago were normalised to deaths per 100,000 of population using census data for the specified year: Kalkstein and Smoyer (1993) - 1970 census, population 3366957. Kalkstein and Greene (1997) - 1980 census, population 3005072. Greene et al. (2011) - 2000 census, population 2896000. Petkova et al. (2014) - 2010 census, population 2707120. Hayhoe et al. (2011) quoted mortality as deaths per 100,000 and so their results are shown without any modification. (PDF) [file pone.0180369.s004.pdf]
