# Supplementary material for: The use of climate information to estimate future mortality from high ambient temperature: A systematic literature review
Source: PLoS One. 2017 Jul 7;12(7):e0180369. doi: 10.1371/journal.pone.0180369 (PMC5501532; doi:10.1371/journal.pone.0180369)
Supplement: S1 Appendix — (PDF) [file pone.0180369.s005.pdf]

S1 Appendix: Calculation of metrics combining temperature and humidity.

S1.1 Apparent Temperature

A commonly used metric for assessing mortality is apparent temperature (AT), which is a function of temperature and humidity. Different formulae have been used to calculate AT. The version most commonly used is shown below:

$$AT = -2.653 + 0.994 \times T_{air} + 0.0153 \times (T_d)^2$$

where T_{air} is the air temperature and T_d is dew point temperature, both with units of °C. Daily mean and daily maximum temperatures have been used to calculate a corresponding daily mean and daily maximum apparent temperature.

S1.2 Humidex:

The humidex ("humidity Index", abbreviated to HX in the present study) is a measure of the combined effect of heat and humidity on human physiology. It is calculated from air temperature and relative humidity. First, the vapour pressure of water v (in hPa) is calculated using:

$$v = (6.112 \times 10\hat{}(7.5*T/(237.7 + T)) * RH/100)$$

where T = air temperature (°C) and RH is the relative humidity (%). The Humidex (HX) is then found using:

$$HX = T + (v - 10) * 5 / 9$$

The Humidex is equivalent to the dry temperature in degrees Celsius. For example, if the dry temperature is 25°C and the Humidex is 30, the humid heat would feel like a dry temperature of 30°C. According to *Environment and Climate Change Canada*, A Humidex of less than 29 means no discomfort; 30 to 39 some discomfort; 40 to 45 great discomfort and avoid exertion; and above 45 is dangerous, heat stroke is possible.

S1.3 Heat Index

The heat index (HI) should not be confused with the Humidex. The HI is also calculated from air temperature and relative humidity. The equation consists of terms up to the third power in both air temperature and humidity, including cross-terms. A full description of the heat index and its calculation is given by Robinson [1].

1. Robinson PJ. 2001. On the Definition of a Heat Wave. J Appl Meteor 40:762-775.
